# Chitosan-Based Biomaterials for Hemostatic Applications: A Review of Recent Advances

**DOI:** 10.3390/ijms241310540

**Published:** 2023-06-23

**Authors:** Daniela Gheorghiță, Horațiu Moldovan, Alina Robu, Ana-Iulia Bița, Elena Grosu, Aurora Antoniac, Iuliana Corneschi, Iulian Antoniac, Alin Dănuț Bodog, Ciprian Ionuț Băcilă

**Affiliations:** 1 Faculty of Material Science and Engineering, University Politehnica of Bucharest, 313 Splaiul Independentei, District 6, 060042 Bucharest, Romania; daniela.mgm8@gmail.com (D.G.); alinarobu2021@gmail.com (A.R.); anaiulia.bita@gmail.com (A.-I.B.); elena_grosu@yahoo.com (E.G.); antoniac.aurora@gmail.com (A.A.); iulicorneschi07@gmail.com (I.C.); 2 Faculty of Medicine, “Carol Davila” University of Medicine and Pharmacy, 050474 Bucharest, Romania; h_moldovan@hotmail.com; 3 Department of Cardiovascular Surgery, Clinical Emergency Hospital Bucharest, 014461 Bucharest, Romania; 4 Academy of Romanian Scientists, 54 Splaiul Independentei, 050094 Bucharest, Romania; 5 Faculty of Medicine and Pharmacy, University of Oradea, 10 P-ta 1 December Street, 410073 Oradea, Romania; 6 Faculty of Medicine, Lucian Blaga University of Sibiu, 10 Victoriei Boulevard, 550024 Sibiu, Romania; ciprian.bacila@ulbsibiu.ro

**Keywords:** hemostasis, topical hemostatic agents, chitosan-based composites, blood–material interaction

## Abstract

Hemorrhage is a detrimental event present in traumatic injury, surgery, and disorders of bleeding that can become life-threatening if not properly managed. Moreover, uncontrolled bleeding can complicate surgical interventions, altering the outcome of surgical procedures. Therefore, to reduce the risk of complications and decrease the risk of morbidity and mortality associated with hemorrhage, it is necessary to use an effective hemostatic agent that ensures the immediate control of bleeding. In recent years, there have been increasingly rapid advances in developing a novel generation of biomaterials with hemostatic properties. Nowadays, a wide array of topical hemostatic agents is available, including chitosan-based biomaterials that have shown outstanding properties such as antibacterial, antifungal, hemostatic, and analgesic activity in addition to their biocompatibility, biodegradability, and wound-healing effects. This review provides an analysis of chitosan-based hemostatic biomaterials and discusses the progress made in their performance, mechanism of action, efficacy, cost, and safety in recent years.

## 1. Introduction

### 1.1. Hemorrhage in Surgical and Trauma Setting

Hemorrhage is a life-threatening condition that represents the first most common cause of death in combat casualties and the second among civilians as a result of traumatic injury. Hemorrhage refers to excessive blood loss occurring due to the unsuccessful formation of a platelet plug at the site of injury [1,2].

In traumatic injuries, bleeding represents a leading cause of potentially preventable death. Moreover, trauma-induced coagulopathy is a complication attributed to trauma that describes abnormal clotting processes in which blood clots are not formed properly. Coagulation abnormalities are associated with internal bleeding and require proper management to restore circulating blood volume and reduce the risk of worsening trauma-induced coagulopathy [1,3]. Efficient bleeding management in the first hour of post-injury is the key to minimizing the deleterious side effects of uncontrolled bleeding [2,4].

**Figure 1:**
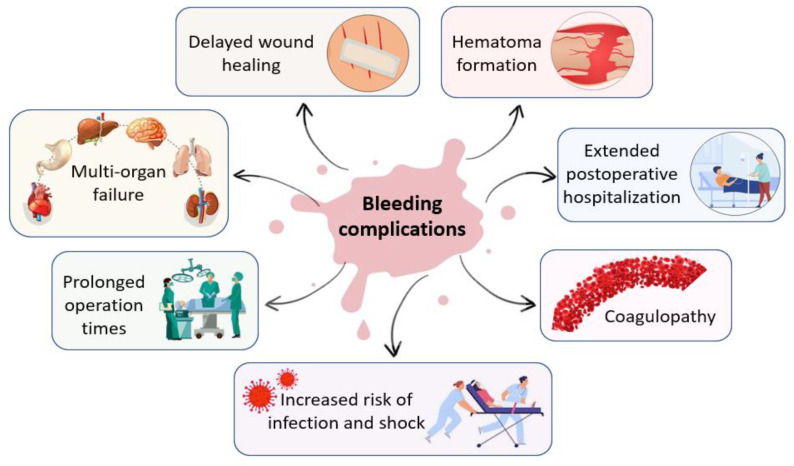
Main complications of uncontrolled bleeding in the intraoperative environment.

Additionally, many complex surgical interventions, such as cardiovascular, cranial, spinal, orthopedic, and liver surgeries present a high incidence of uncontrolled bleeding that require hemostatic intervention [5]. In the intraoperative environment, uncontrolled bleeding can cause a wide array of complications for both surgeons and patients that may lead to adverse intraoperative or perioperative outcomes, including prolonged operation times and postoperative hospitalization, delayed wound healing, increased risk of infection and shock, hematoma formation, multi-organ failure, coagulopathy and increased morbidity and mortality [2,6,7,8]. Figure 1 presents the complications of uncontrolled bleeding.

During surgery, it is important to achieve rapid and effective hemostasis to retain visualization of the surgical field, maintain patients’ hemodynamic equilibrium, and reduce procedure and anesthesia time as well as the occurrence of complications. Bleeding that is not effectively controlled can cause important blood loss volumes, which may require blood transfusions or blood-related products, thus exposing the patient to the numerous complications associated with transfusions, such as immunologic reactions, infection, and immunosuppression, thereby complicating the operative procedure and increasing the risk of morbidity and mortality [9,10,11].

Preventing excessive blood loss during surgery equates to significantly decreasing the risk of major perioperative complications. Therefore, as the risk of surgical re-intervention decreases, the patient needs a shorter hospital stay, which in turn may result in lower hospitalization costs. The appropriate management of such cases is critically important and thus a major consideration for surgeons aiming to avoid these adverse effects [7,11,12].

### 1.2. Achieving Hemostasis

Hemostasis represents the body’s natural and physiological reaction to injury and the first step in wound healing. It is a vital process that involves multiple interlinked steps in order to stop the bleeding by forming a stable clot. The mechanism of hemostasis works like a multifaceted response to prevent and stop the blood loss that occurs due to a disruption of the vessel walls [1,13]. 

**Figure 2:**
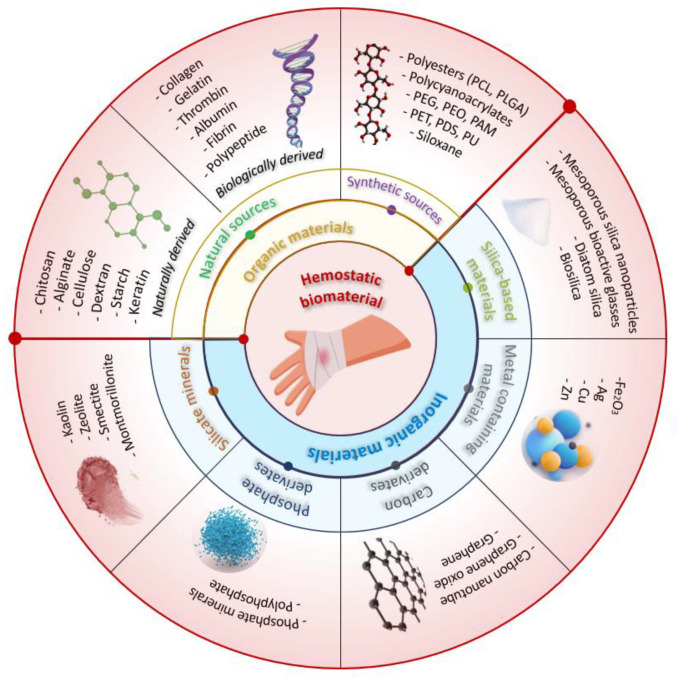
Schematic illustration of multiple external hemostatic materials for hemorrhage control.

Conventional techniques for achieving hemostasis in a surgery setting include a variety of mechanical techniques (e.g., sutures, ligatures, vascular clips, bone wax) and thermal techniques (e.g., electrocautery). To further aid in achieving hemostasis, adjunctive topical hemostatic products are also employed in conjunction with these primary techniques. Topical hemostatic agents play an important role in the surgery environment as well as in first-aid treatment, controlling blood loss and minimizing the risk of associated complications and consequent mortality and morbidity [2]. Figure 2 schematically illustrates multiple topical hemostatic materials used to control hemorrhage.

**Figure 3:**
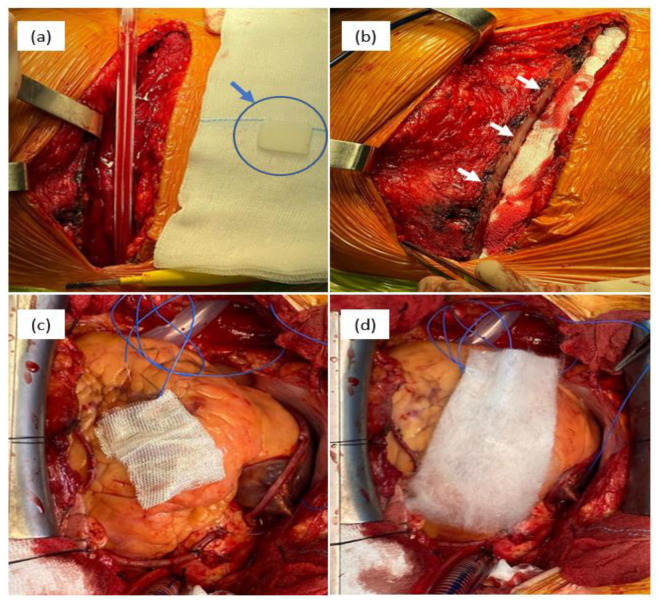
Intraoperative aspects of different hemostatic materials used in cardiovascular surgery: bone wax—(**a**) physical aspect of bone wax (blue arrow) and (**b**) sternum aspect after the application of bone wax (white arrows); (**c**) cellulose-based material, type Surgicel Original; and (**d**) cellulose-based material, type Surgicel Fibrillar.

Topical hemostatic agents can be categorized based on the raw materials used into two types: organic-based and inorganic-based hemostatic products [14]. Organic-based hemostatic agents have two sources: natural sources that comprise both naturally derived (carbohydrate-based) and biologically derived (protein-based) materials and synthetic sources. Figure 3 presents the intraoperative aspects of different hemostatic materials used in cardiovascular surgery.

Polysaccharides such as chitosan, alginate, cellulose, dextran, starch, and keratin are currently the most commonly used natural polymeric hemostatic materials that are widely available. While biologically derived hemostatic materials (e.g., collagen, gelatin, thrombin) facilitate platelet aggregation and activation, achieving rapid hemostasis, their high cost, risk of immune reactions, and poor mechanical properties have limited their widespread use [15]. Many researchers have aimed to develop hemostatic agents from natural resources and the results have proven to be promising. For example, Singh Chandel et al. conducted a comprehensive investigation to assess the impact of sponge compression on the hemostatic and antiadhesive properties of bilayer alginate sponges prepared through lyophilization. The findings of their study revealed that the 100 mm compressed sponge exhibited enhanced hemostatic effects in a mice liver bleeding model, whereas the 200 mm compressed sponge displayed improved antiadhesive properties in a rat model of hepatectomy-induced adhesion [16]. A novel hemostatic sponge composed of chemically cross-linked gelatin, developed by Reiner Hajosch et al., demonstrated promising results. The gelatin sponge, manufactured using pharmaceutical-grade materials, exhibited exceptionally fast absorption of human blood based on in vitro blood uptake assays. The absorption rate was found to be two to three times quicker compared to other hemostatic devices tested. Furthermore, in an in vitro hemorrhage model utilizing human veins, the novel gelatin sponge matrix achieved hemostasis within less than a minute following the induction of bleeding [17].

Synthetic hemostatic agents are represented by polyesters (polycaprolactone (PCL), poly (lactic-co-glycolic acid) (PLGA)), poly(ethylene glycol) (PEG), polycyanoacrylates, polyurethane (PU), siloxane, polyethylene oxide (PEO), polyacrylamide (PAM), polyethylene terephthalate (PET), and polydioxanone (PDS). These agents are widely used in various hemostasis operations because of their low immunogenicity, relatively high stability, and the ability to customize their chemical properties to enhance their clinical performance. However, the production cost of synthetic polymers is usually higher than that of natural polymers, and their poor biodegradability and potential cytotoxicity may impede their use in clinical practice [18].

Several inorganic materials have been developed to accelerate blood coagulation, including silicate minerals, silica-based materials, metal-containing materials, phosphate, and carbon derivatives. Inorganic hemostatic materials are often cheaper than organic materials, and they are physically and chemically stable, easy to produce and transport, and carry no risk of bloodborne disease. However, most inorganic materials are not bioabsorbable and need to be removed after use.

Some types of natural mineral clays (silicate minerals) have been found to have hemostatic properties and are used in medical applications to promote hemostasis. Kaolin is a type of clay that is commonly used in medical applications to promote hemostasis. It has a large surface area and can absorb water and blood, which helps to promote clotting. Montmorillonite is a type of clay that has a layered structure and a high cation exchange capacity. It is often used in hemostatic dressings and can promote clotting by absorbing blood and concentrating platelets. Zeolites are microporous, crystalline aluminosilicates that have unique properties, including a high surface area and the ability to selectively adsorb molecules [19,20,21].

Phosphates are a class of compounds that contain the element phosphorus. They have been studied for their potential use as hemostatic agents, particularly in the form of calcium phosphate materials. Hydroxyapatite is a common component of bone and has been shown to be effective in promoting hemostasis in surgical settings. Tricalcium phosphate is another calcium phosphate material that has been studied for its hemostatic properties and has been shown to be effective in controlling bleeding in dental procedures.

Carbon derivatives are another class of compounds that have been investigated for their potential use as hemostatic agents. Carbon nanotubes are long, thin tubes made up of carbon atoms that have unique mechanical and electrical properties. Carbon nanotubes and graphene oxide have been shown to promote blood clotting and enhance the effectiveness of other hemostatic agents [22,23].

Silica-based materials, including mesoporous silica, mesoporous bioactive glasses, diatom silica, and their composites, have recently been explored as a promising avenue in the field of hemostasis due to their negative charge and highly absorptive pores, which give them inherent hemostatic abilities. Diatom silica, for instance, is a nanostructured silica biomaterial with a 3D porous structure with high porosity and specific surface area, enabling it to absorb water, concentrate coagulation factors, and promote hemostasis. Mesoporous silica particles share similarities with natural mineral clays in that they can quickly absorb water and aggregate platelets, blood cells, and coagulation factors at the site of injury [24,25]. Table 1 highlights the characteristics and hemostatic mechanism of different hemostatic materials and commercially available topical hemostatic agents. Table 2 presents examples of commercial brands of hemostatic materials containing chitosan and their mechanism of action.

Metal-containing materials have been effectively utilized in the field of hemostasis due to their demonstrated procoagulant activity. Silver nanoparticles have been shown to have antimicrobial properties, which may be useful in preventing infections at the site of bleeding and promoting blood clotting. Copper nanoparticles have been shown to have antibacterial properties and promote blood clotting in both in vitro and in vivo experiments. Similar to copper nanoparticles, zinc nanoparticles have been found to promote blood clotting and prevent bleeding when applied to a wound. Several studies showed that zinc nanoparticles significantly accelerated blood clotting in vitro and demonstrated antibacterial activity [68,69,70,71,72,73].

There is a significant range in the cost of different agents, with pricing observed to be influenced by the active ingredient responsible for stopping bleeding. Among the less costly hemostatic agents were those that are absorbable and composed of oxidized regenerated cellulose, chitosan, collagen, and gelatin, with a price range between $15 and $100. Absorbable agents made with microfibrillar collagen are priced at a higher level compared to other absorbable agents ($101–$300). Biologically active agents (made with human fibrinogen and human thrombin), dual agents (combining thrombin and gelatin), and synthetic hemostatic agents range from $301 to $500, with the latter being the most expensive [74,75]. For example, Baxter Coseal Surgical Sealant, which consists of two biocompatible polyethylene glycols that form a covalently bonded hydrogel (adhering to both tissue and synthetic graft materials), has a price of $439.00 per kit [76,77], while a 15 g pack of Celox Haemostatic Granules, consisting of macroscopic granular flakes prepared from chitosan, costs only $16.67 [78]. Thus, chitosan is more cost-effective compared to other alternatives [66].

According to the analysis conducted by Data Bridge Market Research, the hemostats market is projected to reach USD 4.65 billion by 2029, with a compound annual growth rate (CAGR) of 6.85% during the forecast period. The market growth is primarily driven by the rising number of surgical procedures being performed. The largest share in the hemostatic market is attributed to the thrombin-based hemostats segment when categorized by type. When considering formulation, the matrix and gel segment held the largest share. The medical-grade chitosan market was valued at USD 284.6 million in 2022, and it is projected to reach USD 610.2 million by 2030. The market is expected to grow at a compound annual growth rate (CAGR) of 10% during the period from 2022 to 2030. In 2022, the shrimp segment held the largest revenue share, accounting for 63% of the chitosan market. Shrimp is the primary source of chitosan due to its higher chitin content, ranging from 25% to 40%, compared to crab shells, which typically contain around 15% to 20% chitin. Two of the major players in the chitosan market are Primex EHF, an Icelandic marine biotechnology company renowned for its sustainable production of high-quality chitosan, and Heppe Medical Chitosan GmbH, a German chitosan producer catering to the global medical technology and pharmaceutical industry [79,80].

More research is needed to fully understand their mechanisms of action and to optimize their use in hemostasis. It is also important to consider potential safety concerns associated with the use of these materials in medical applications, including the potential for toxicity or immunological reactions [81,82]. Numerous materials possess inherent properties that assist with the coagulation process and can effectively act as hemostatic agents. However, complex clinical requirements are not always met by the hemostatic efficiency of these materials.

When evaluating the efficacy of hemostatic agents, several clinical endpoints can be considered. These include parameters such as the time to hemostasis (the time it takes for bleeding to stop), the reduction in blood loss or volume, the improvement in clot formation or stability, the prevention of rebleeding, wound healing, and overall patient outcomes such as morbidity and mortality rates. The specific endpoint results should be evaluated according to the specific context and clinical situation.

Although the desired properties of an ideal local hemostatic agent may differ depending on the surgical specialty, certain characteristics are generally valued, including immediate and effective bleeding control, good safety profile (non-toxic, non-immunogenic), and ease of preparation and administration [9]. 

The process of blood clotting involves a complex interplay of biological, chemical, and physical reactions. In various blood-clotting mechanisms, chitosan has demonstrated its effectiveness in hemostasis by virtue of its porous structure. By working in tandem with two or more hemostatic mechanisms, the hemostatic efficacy of chitosan can be further enhanced [65].

## 2. Chitosan Properties and Hemostasis Efficiency

### 2.1. Chitosan Source and Structure

Chitin is a white, hard, inelastic, nitrogenous, natural polysaccharide (molecular formula: (C_8_H_13_O_5_N)_n_), which was extracted in 1911 from mushrooms and has been identified as the second-most abundant polysaccharide found in nature after cellulose [2,66,83]. Chitin represents a strengthening material for the cell walls of fungi, the exoskeletons of crustaceans (e.g., shrimps, lobsters, crabs) and insects, and fish scales [83,84,85]. Chitin and its derivatives are used in various sectors such as chemistry, cosmetics, medicine, and agriculture as well as in the textile and paper industry [84]. For biomedical applications, chitin is usually converted through biological (enzymatic) or chemical deacetylation to its most well-known derivative, chitosan [83]. Microorganisms and enzymes are used in biological methods, but the main obstacle to achieving scalable enzymatic deacetylation is the high crystallinity of chitin. Chemical deacetylation, performed under severe alkaline conditions, is the predominant method used for the industrial-scale conversion of chitin [86,87].

When considering the desired application of chitosan, the source and obtention process play crucial roles as they determine the characteristics of the final product. For biomedical purposes, factors such as purity, crystallinity, molecular weight (Mw), and deacetylation degree (DD) are particularly significant. These factors are closely linked to the mechanical and biological properties of chitosan [86,88].

**Figure 4:**
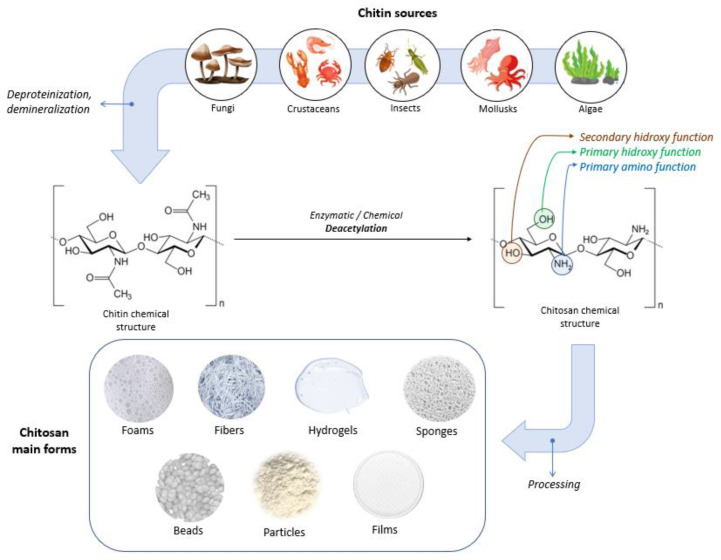
Sources, chemical structures of chitin and chitosan, and chitosan’s main forms.

Chitosan can have varying degrees of deacetylation and is classified as a copolymer of N-acetyl-D-glucose amine and D-glucose amine (C_6_H_11_O_4_N)_n_ containing a varied number of N-acetyl groups [66,84]. Depending on the source and preparation, the molecular weight of chitosan is typically between 300 and 1000 kDa, with a DD varying between 60 and 100% [89]. The acetylation degree of chitosan is at least 60% of glucose amine residue [88,89]. The high versatility of chitosan offers the possibility to be used in physical forms such as fibers (and nanofibers), films and dressings, gels and hydrogels, sponges, beads, particles (and nanoparticles), membranes, and scaffolds [86]. Figure 4 illustrates sources, the chemical structures of chitin and chitosan, and chitosan’s main forms.

### 2.2. Chitosan Properties

Chitosan is a weak base insoluble in H_2_O and organic solvents but soluble in acidic solutions (pH < 6.5) [83,90]. Due to its increased versatility and biological properties, much research has been conducted on chitosan. In vitro studies have demonstrated that chitosan exhibits superior cytocompatibility when compared to chitin [84].

Chitosan is a biopolymer that has captured the attention of researchers and industries alike due to its unique physical and chemical properties, remarkable macromolecular framework, and biological activities. These attributes distinguish it from synthetic polymers, making it an exciting and promising material for applications in fundamental science, applied research, and industrial biotechnology [91]. 

Significant attention is directed towards its potential medical and pharmaceutical applications due to its remarkable properties, such as biocompatibility, biodegradability, and non-toxicity, which make it very valuable in the biomedical field [84,92]. Chitosan is a biocompatible substance that does not trigger an immune response, making it compatible with living tissues. Chitosan also possesses several other distinctive properties, including hemostatic and antithrombogenic properties and the ability to form polyoxysalts, create films, and demonstrate molecular adsorption properties [83]. Several studies have shown that chitin and chitosan are both biocompatible and biodegradable biopolymers, present antimicrobial properties, and enhance blood coagulation [90,93]. 

The bacteriostatic and fungistatic properties of chitosan-based materials are particularly useful for wound treatment. In addition to their antimicrobial properties, chitosan and its oligosaccharides can stimulate cell growth. Chitosan-based materials such as non-wovens, nanofibers, composites, films, and sponges have been shown to promote wound healing and dermal regeneration. As a result, chitosan’s primary commercial applications in the biomedical field are related to wound healing [94]. 

Due to its versatility, chitosan has proven to be a valuable material for a variety of practical applications in numerous fields such as medicine, chemistry, cosmetics, biotechnology, agriculture, and chromatography, as well as textile and fiber industries [95]. Chitosan has tremendous potential in the medical and biomedical fields, with applications ranging from pharmaceutical formulations and drug delivery (including antibiotics, vaccines, anti-inflammatory agents, peptides, proteins, and growth factors) to antimicrobial treatments, burns, wound healing, gene delivery and therapy, regenerative medicine, and tissue engineering (for tendon, cartilage, ligament, bone, liver, neural, and skin regeneration). Additionally, chitosan has potential applications in cancer treatment, therapy, and diagnostics, as well as in dentistry, dermatology, ophthalmology, biosensors, bio-imaging (such as magnetic resonance imaging), support for immobilized enzymes, and veterinary medicine [84,94,96]. 

The widespread use of chitosan is somewhat restricted due to its limitations, including poor solubility, reactivity, and certain physical properties such as rigidity and brittleness. Chitosan, in its pure form, is insoluble in aqueous solutions and exhibits limited antibacterial activity when dissolved. Additionally, the poor solubility of unmodified chitosan in organic solvents further restricts its practical applications [86].

Chitosan’s molecular structure possesses three distinct types of reactive functional groups: a secondary hydroxyl group at C-3, a primary hydroxyl group at C-6, and an amino group at C-2, as displayed in Figure 4. The presence of these functional groups enables the conjugation of various substituents, leading to the development of new modified derivatives of chitosan.

Some of the modifications include nitration, sulfation, thiolation, acylation, esterification, carboxyalkylation, phosphorylation, graft copolymerization, and crosslinking techniques [97]. The principal derivatives of chitosan include carboxymethyl chitosans, quaternary ammonium chitosan salts, carboxyalkyl chitosans, hydroxyalkyl chitosans, phosphorylated chitosan, and thiolated chitosans. Among these derivatives, carboxymethyl chitosans have been extensively studied and explored [94,97].

The modifications applied to chitosan provide it with new properties. For example, the introduction of quaternary ammonium groups into chitosan increases the number of positively charged centers, resulting in enhanced platelet aggregation compared to chitosan alone. Additionally, it has been observed that platelet aggregation increases with the degree of substitution of these groups [98]. In a study conducted by Wang et al., a novel hemostatic hydrogel was developed by chemically reacting the carboxyl group in carboxymethyl chitosan. The resulting hydrogel exhibited favorable mechanical strength and swelling properties. In vitro coagulation assay results indicated that the hydrogel had the potential to enhance blood clotting at wound sites [99].

### 2.3. Hemostatic Application of Chitosan

The concept of blood coagulation originated in the 1960s when “waterfall” and “cascade” theories of blood coagulation were proposed, serving as a cornerstone for the exploration of endogenous coagulation pathways. 

Numerous studies [100,101] have demonstrated that chitosan induces coagulation without activating the intrinsic pathway, suggesting that the hemostatic mechanism of chitosan operates independently of the classical coagulation cascade. This aspect makes chitosan an ideal biomaterial of particular interest because it can stop bleeding in coagulopathic patients (clotting dysfunction) [66,101].

While the specific hemostatic mechanism of chitosan is not yet fully comprehended, available data indicate some potential ways in which it may help regulate bleeding [100]. Thus, some properties of chitosan (cationic, absorbent) are of great importance for promoting hemostasis.

While many naturally occurring polysaccharides, such as agar, dextran, pectin, carrageenan, cellulose, agarose, and alginic acid are neutral or acidic in nature, chitosan stands out as an example of a highly basic polysaccharide. Chitosan’s most distinctive property is its cationic nature and its unique behavior in solution, which is also of great importance for its medical applications. This biopolymer is the only naturally occurring cationic polymer known to exist in nature [94,102].

**Figure 5:**
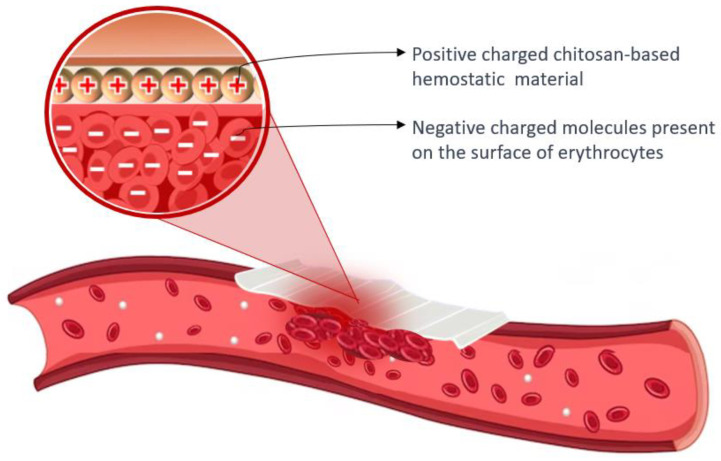
Hemostatic mechanism of chitosan-based material: the aggregation of erythrocytes occurs due to the interaction between positively charged chitosan and negatively charged molecules present on the surface of erythrocytes.

Chitosan’s cationic properties play a significant role in inducing hemostasis, as the surfaces of erythrocytes carry negative charges. The presence of opposite charges between chitosan and erythrocytes leads to the assertion that chitosan has an attracting effect and crosslinks with erythrocytes, resulting in the formation of a “mucoadhesive barrier” at the wound site to halt bleeding [101,103]. The amino groups found in chitosan (poly-N-acetyl glucosamine) play a crucial role in facilitating the aggregation of erythrocytes through electrostatic interactions with the surface charges of the cells [66,89]. Figure 5 presents the hemostatic mechanism of chitosan-based material.

Moreover, this cationic nature of chitosan also allows it to bind with the anions on bacterial cell walls, thereby impeding their entry into the cell. This microbicidal action of chitosan is crucial in the wound-healing process [96]. It is noteworthy that the majority of the biological and chemical applications of chitosan rely on its cationic properties and its versatility as a biomaterial [94].

Upon contact with blood, chitosan has been demonstrated to stop bleeding by absorbing liquid, which leads to the concentration of erythrocytes and platelet adherence to the injured area [84]. The molecular weight and degree of deacetylation attained during the purification process have a significant impact on the hemostatic efficacy of chitosan. A higher degree of deacetylation enhances the aggregation of erythrocytes and platelets, which is crucial for initiating hemostasis [66].

In fact, scientific research has shown that a chitosan dressing was able to effectively control an arterial hemorrhage in dogs [104]. Several animal model studies have demonstrated that chitosan-based dressings are effective hemostatic agents, even in coagulopathic conditions. Chitosan has been demonstrated to be a highly effective hemostatic agent in various formulations such as gels/hydrogels, powders, membranes and films, dressings, sponges, foams, and microspheres. 

## 3. Current Trends in Chitosan-Based Hemostatics

There is a growing demand for biomaterials that can perform multiple functions, including hemostasis, antibacterial activities, the stimulation of cell division and differentiation, and the acceleration of tissue healing. 

The composite dressing was found to possess high porosity, allowing it to absorb a significant amount of blood while also promoting hemostatic activity through platelet aggregation and activation. Additionally, the dressing exhibits antibacterial properties against *E. coli* and *S. aureus*. The incorporation of ibuprofen drugs, nano zinc oxide, gallic acid, and cinnamaldehyde has further enhanced the antimicrobial and hemostatic properties of chitosan-based materials [65].

A study conducted by Pan et al. [105] demonstrated that the incorporation of zinc alginate in chitosan microspheres resulted in the activation of coagulation factor XII and the initiation of the intrinsic coagulation cascade. This led to the production of thrombin and fibrin clots, as well as the acceleration of blood coagulation time, clotting, and clot propagation, in addition to fibrin clot formation.

Combining chitosan with alginate, cellulose, silk, and Bletilla striata polysaccharide has been shown to induce hemostatic activity by reducing hemostasis time and minimizing blood loss. This approach can effectively manage bleeding complications during surgical procedures in diabetic patients [106,107]. 

The microbiological aspect of hemostatic materials is of great importance. One of the primary concerns when dealing with hemostatic materials is the prevention of infections. Microorganisms, such as bacteria and fungi, can potentially contaminate these materials and lead to infections in the wound site. By considering the microbiological aspect, the design and formulation of hemostatic materials can be optimized to minimize the risk of microbial contamination and subsequent infections. Moreover, microorganisms present in or around the wound can significantly impact the healing process. Certain microorganisms can hinder wound healing, cause complications, or lead to chronic infections. Understanding the microbiological aspects of hemostatic materials allows for the development of products that can not only control bleeding but also promote an environment conducive to wound healing by preventing microbial colonization or supporting antimicrobial activity [108].

In recent years, the application of nanotechnology in the biomedical field has led to the development of new tools. For instance, incorporating silver nanoparticles (AgNPs) into chitosan-based hydrogels has been proposed as a means of introducing a bactericidal and bacteriostatic agent [108]. According to a report by Vijayakumar and colleagues in 2019, chitosan–AgNPs hydrogels demonstrated superior antibacterial activity when compared to uncoated hydrogels. The in vitro antibacterial activity of the hydrogels was evaluated against wound infections caused by methicillin-resistant *S. aureus* and *P. aeruginosa*. The results showed that chitosan–AgNPs hydrogels exhibited effective antibacterial action [108].

Different forms of chitosan-based materials for hemostatic application and their characteristics findings are presented in Table 3.

### 3.1. Dressing

Hemostatic dressings are designed to support or collaborate with the body’s innate clotting mechanism to control bleeding. It has been reported that chitosan-based dressings can expedite hemostasis, even in the presence of coagulopathy [120,121].

Santosh S. Biranje et al. conducted a study in which a porous chitosan nanoparticulate dressing was prepared using the lyophilization method for potential use in wound healing. The chitosan dressing exhibited high porosity, improved swelling properties, controlled biodegradation, and biocompatibility, as well as accelerated hemostatic activity. These results suggest that chitosan-based dressings have immense potential for use in wound-healing applications [109]. 

In their study, Wang et al. obtained chitosan/polybutylenes succinate (CS/PBS) nanofiber membranes with varying CS contents of 0%, 20%, 40%, 60%, 80%, and 90% using the electrospinning technique. The results of the research demonstrated that the CS/PBS fiber membrane with different CS contents exhibited distinct characteristics such as varying fiber diameter, wettability, liquid absorption, moisture permeability, and blood-clotting performance. The membrane with a CS content of 90% demonstrated the most favorable properties for wound dressing, making it the most suitable option [122].

In a study developed by Buriuli et al., a dressing was obtained from chitosan and pectic acid using sonication and freeze-drying methods. The dressings featured a porous structure, exceptional water and blood absorption, strong hemostatic performance, and a lightweight and flexible structure, making them a promising material for potential use as a hemostatic agent [120].

Akram et al. obtained a chitosan/calcium phosphate (CCP) hemostatic dressing that was found to be effective in controlling blood loss and reducing blood-clotting time to only 15 s. Moreover, the CCP-based hemostatic dressings were found to be effective in controlling bacterial infections [123].

**Figure 6:**
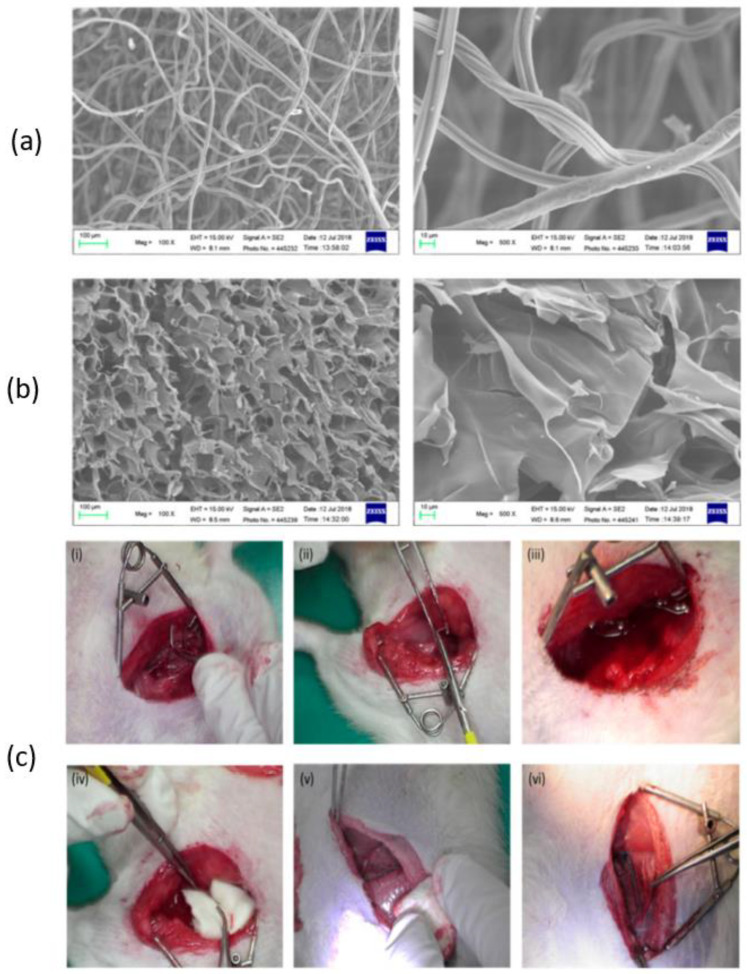
SEM analysis of (**a**) chitosan fiber, (**b**) chitosan sponge, and (**c**) the in vivo assessment of the hemostatic efficacy of dressings tested on a rat femoral artery hemorrhage model. Figure is licensed under CC-BY 4.0 [124].

According to Wang et al., chitosan fiber (CF) dressing exhibited stronger hemostatic properties compared to regular gauze-type surgical dressing. In an animal model of femoral artery hemorrhage and in patients with surgical wounds, CF dressing reduced the hemostasis time and effectively controlled blood loss and absorption [124]. Figure 6 presents a scanning electron microscopy (SEM) analysis of chitosan-based dressings and the rat femoral artery hemorrhage model that was used to evaluate the dressings. The surface of the CF dressing appeared to be evenly fibrous, with fibers measuring approximately 10 μm in diameter, as shown by SEM in Figure 6a. On the other hand, the chitosan sponge exhibited a highly porous surface, with pore diameters ranging from around 50–100 μm (Figure 6b). These dressings were both characterized by large surface areas and interconnected networks, with the CF dressing having the highest surface area among the two. In this study, a rat femoral artery hemorrhage model was employed to assess the effectiveness of the dressings. This model simulates a severe injury to the groin region, which results in partial damage to the femoral artery and life-threatening bleeding that cannot be managed by standard dressings (Figure 6c).

**Figure 7:**
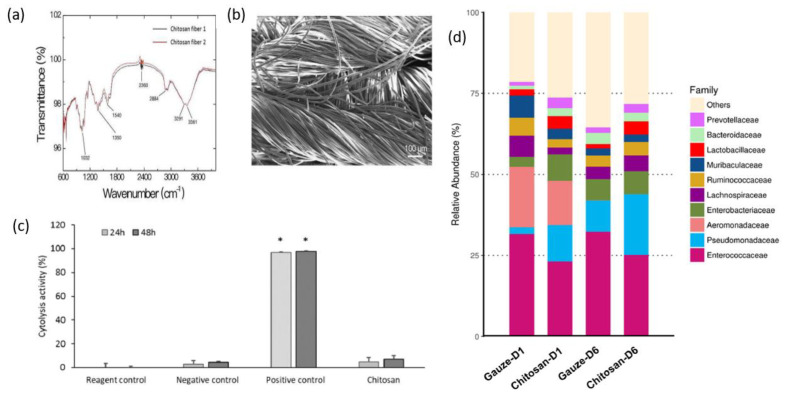
Characterization of the chitosan dressing: (**a**) Fourier-transform infrared spectroscopy results; (**b**) morphology observations using scanning electron microscopy; (**c**) cytolysis activity measurement for biocompatibility evaluation (* *p* < 0.05; 24 and 48 h incubation); (**d**) relative abundance of most predominant bacteria identified in wound-contact chitosan and regular gauze dressings up to 6 days post-surgery (D1 = day 1, D6 = day 6). Figure is licensed under CC-BY 4.0 [125].

The findings of C.-H.Wang et al. showed that chitosan dressing outperforms regular gauze-type surgical dressing in terms of antimicrobial and procoagulant properties when applied to surgical wounds in patients. The study suggests that chitosan dressing not only possesses antimicrobial and procoagulant properties but also has the potential to promote wound healing by introducing beneficial microbiota [125]. Figure 7 presents some results after the characterization of the chitosan dressing, including Fourier-transform infrared spectroscopy (FT-IR) results, scanning electron microscopy (SEM) observations, cytolysis activity measurements, and relative abundance of most predominant bacteria identified in wound contact up to 6 d post-surgery. Fourier-transform infrared (FTIR) spectroscopy analysis, as presented in Figure 7a, demonstrated the characteristic transmittance bands of chitosan dressing. Scanning electron microscopy (SEM) analysis, shown in Figure 7b, demonstrated that the chitosan dressing had a uniform fibrous structure and a significantly large surface area. The chitosan dressing showed a high level of biocompatibility as it was able to significantly inhibit cytolysis (Figure 7c), similar to the negative control represented by Dulbecco’s modified Eagle’s medium (DMEM) and phosphate-buffered saline (PBS). The study utilized 16S rRNA-based sequencing on the Illumina MiSeq platform to analyze the microbial community in the wound affected by the dressing treatments. Figure 7d illustrates the effect of wound dressings on the relative abundance of the bacterial population at the family level. The results showed that the chitosan dressing was able to suppress the growth of Enterobacteriaceae members (from 8.1% in 1 day to 7% in 6 days post-surgery) while promoting the growth of Pseudomonadaceae members by 7.6% up to 6 days post-surgery compared to the regular gauze dressing.

### 3.2. Hydrogel

Hydrogels have gained attention for clinical applications due to their ability to maintain a moist environment and seal tissue during hemostasis, thanks to their high-water content. They are composed of hydrophilic polymers cross-linked in a three-dimensional network that closely resembles the extracellular matrix, allowing them to absorb substantial amounts of water. The ability of hydrogels to swell is attributed to the presence of hydrophilic groups (-OH, -CONH-, -CONH_2_, and -SO_3_H) within the polymeric components of the gels [126,127].

In a study conducted by Qiao et al., (2021), a supramolecular hydrogel was developed by combining chitosan with silk fibroin and using tannic acid as a crosslinker. The resulting hydrogel exhibited a strong wet adhesion capacity and demonstrated rapid hemostatic activity in various arterial and visceral bleeding models. These findings suggest that the use of supramolecular hydrogels could be a promising strategy for achieving hemostasis in clinical applications [14].

**Figure 8:**
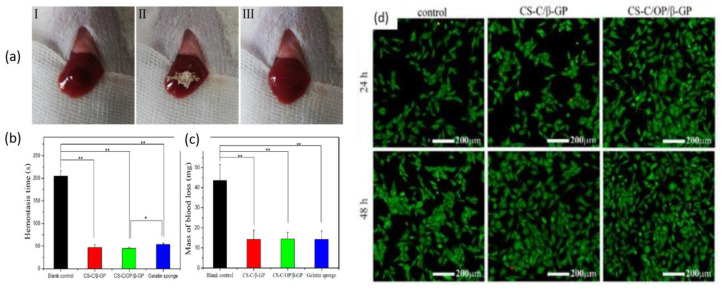
Hemostasis in a liver injury model (I: bleeding; II: hemostasis; III: aspect of the wound after hemostasis): (**a**) time of hemostasis; (**b**) mass of blood loss; (**c**) and Calcein-AM/PI double staining for L929 cells, * *p* < 0.05 and ** *p* < 0.01; (**d**). Figure is licensed under CC-BY 4.0 [128].

Zhang et al. prepared chitosan-based thermo-sensitive hydrogel loading oyster peptides (CS-C/OP/β-GP) using catechol-modified chitosan (CS-C) as the matrix material and β glycerol phosphate (β-GP) as a thermo-sensitive agent. According to the findings, the coagulation time and blood coagulation index of the CS-C/OP/β-GP hydrogel were superior to those of a commercially available gelatin sponge when tested in vitro [128]. Moreover, the platelet adhesion and erythrocyte adsorption rates of the CS-C/OP/β-GP hydrogel were significantly higher, showing an increase of 38.98% and 95.87%, respectively, when compared to the gelatin sponge. Furthermore, it was observed that the use of CS-C/OP/β-GP hydrogel reduced the hemostasis time in mouse liver injury by 19.5% and decreased the mass of blood loss in the mouse tail amputation model by 18.9%. Safety evaluations also revealed that the CS hydrogel was non-cytotoxic to L929 cells, and the hemolysis rates were under 5% at concentrations of up to 1 mg/mL, indicating favorable biocompatibility. These findings suggest that CS hydrogel has the potential to be a reliable medical dressing for hemostasis, making it a promising candidate in this field. Figure 8 shows the application of samples in the hemostasis of liver injury (a), liver hemostasis time (b), liver blood losses (c), and Calcein-AM/PI double staining for L929 cells (d). Figure 8d illustrates that there was no notable variation between the control group and the samples after 24 h. However, CS-C/β-GP and CS-C/OP/β-GP exhibited intense green fluorescence when compared to the control group after 48 h. Upon visual inspection of the samples, it was observed that aside from a few dead cells, the majority of the cells retained their normal spindle shape, and there was a higher cell density and uniform distribution. These findings suggest that L929 cells were able to grow and develop normally in the presence of CS-C/OP/β-GP. The CS-C/OP/β-GP thermo-sensitive hydrogel possesses a porous three-dimensional network structure that enables it to rapidly absorb water and concentrate blood. The fast and efficient hemostatic properties of hydrogel can be attributed to multiple factors. Firstly, the three-dimensional porous structure of the material plays a significant role. Additionally, chitosan, present in the hydrogel, activates multiple coagulation pathways that are independent of the classical coagulation cascade. Moreover, the oyster peptides present in the hydrogel activate endogenous coagulation factors, further enhancing the hemostatic process. The combination of these factors contributes to the rapid and effective hemostasis achieved by the CS-C/OP/β-GP hydrogel.

G. Patil et al. obtained a novel chitosan-based hydrogel that was loaded with SiNPs and AlCl_3_ and demonstrated its effectiveness as a hemostatic dressing for non-compressible bleeding. The hydrogel composite exhibited improved platelet aggregation and calcium store activation, leading to faster hemostasis. Additionally, the soft texture of the hydrogel allowed for easy application and was determined to be safe [129].

### 3.3. Sponge

Typically, sponges have a porous structure, which makes them permeable to gas exchange, flexible, and able to absorb exudates from skin lesions [130].

Kim and colleagues [131] developed a chitosan–catechol sponge with the aim of improving hemostasis in patients with coagulopathy. They conducted a detailed analysis of the hemostatic mechanism of the sponge and proposed a synergistic mechanism based on the combined action of catechol and cationic charges. This mechanism was found to promote rapid coagulation, even in cases of coagulopathic blood, and was therefore considered promising for clinical application 19. Upon contact with blood, the chitosan-catechol sponge would form a barrier layer by interacting with the proteins in the blood, which helps to prevent bleeding. However, native chitosan has limited film-forming ability since it is not able to dissolve in neutral conditions, which can result in a decrease in its hemostatic efficacy compared to catechol-conjugated chitosan.

In 2020, Wu and colleagues developed a sponge that serves multiple functions, including fast hemostasis and long-term antimicrobial activity [132]. This was achieved by modifying chitosan with mercaptosuccinic acid and using sulfhydryl groups (-SH) to immobilize AgNPs. The introduction of -SH improved the activity of tissue factor (TF), which initiates the coagulation cascade, and also reduced cytotoxicity by slowing down the release rate of AgNPs. Furthermore, the immobilized AgNPs were able to enhance the strength of the formed bleeding clot by serving as action sites for amino, mercapto, and carboxyl groups.

Fan et al. obtained a chitosan/cellulose composite sponge that displayed a high water absorption capacity and mechanical strength [116]. Furthermore, this newly developed sponge also demonstrated inhibitory effects on the growth of *E. coli*, *S. aureus*, and *P. aeruginosa* bacteria. Tests conducted on the chitosan/cellulose composite sponge showed that it had good coagulation ability and demonstrated rapid hemostasis in vivo, as it was able to stop bleeding from a rat leg artery injury in just 34 s.

**Figure 9:**
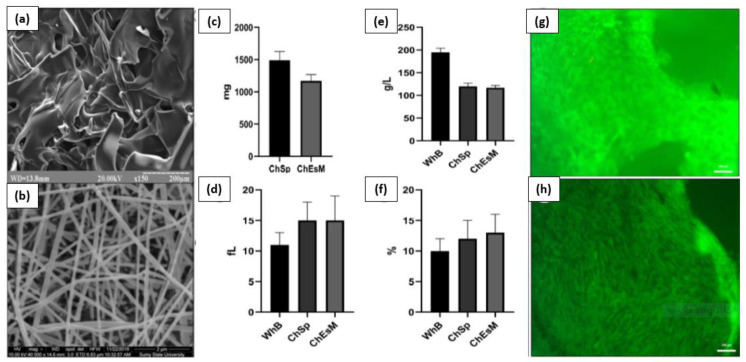
Scanning electron microscopy image of ChSp (**a**) and ChEsM (**b**), blood sorption (**c**), and hematological parameters: platelet (**e**), platelet distribution width (**d**), and mean platelet volume (**f**) after interacting with blood. ChSp (**g**) and ChEsM (**h**) live/dead staining with FDA/PI after 48 h of cell cultivation. Figure is licensed under CC-BY 4.0 [133].

V. Deineka et al. conducted a study to evaluate the biocompatibility, hemostatic efficacy, and tissue-regeneration performance of a Ch-PEO (chitosan–polyethylene oxide) copolymer that was prepared using the electrospinning technique [133]. The chitosan electrospinning membranes (ChEsM) were fabricated from chitosan and polyethylene oxide (PEO) powders to produce a highly porous material with adequate hemostatic properties. The study analyzed the structure, porosity, density, antibacterial characteristics, in vitro degradation, and biocompatibility of ChEsM and compared them with a conventional chitosan sponge (ChSp). In vitro, both materials showed significant biocompatibility and hemostatic efficacy. However, ChEsM exhibited weaker antibacterial properties compared to ChSp. In vivo studies validated the superior biocompatibility and satisfactory hemostatic performance of ChEsM, with close interaction with host tissues and cells. The in vivo model demonstrated a faster biodegradation rate of ChEsM and enhanced liver healing. Figure 9 presents scanning electron microscopy images of the chitosan sponge and chitosan electrospinning membrane, blood sorption, and hematological parameters, as well as live/dead staining with FDA/PI after 48 h of cell cultivation [133].

Bal-Ozturk and colleagues conducted a study in which they fabricated and characterized chitosan/alginic acid/zinc oxide (CHI/AA/ZnO) nanostructured hydrogel sponges for potential use as a hemostatic agent in biomedical applications. The hydrogel sponges containing ZnO demonstrated a good bacteriostatic effect on *S. aureus* in antibacterial experiments, with the antibacterial properties increasing as the amount of ZnO in the polymer network increased [134].

In a study developed by Lin et al., a chitosan–graphene oxide hemostatic sponge was obtained with a porous structure that allows it to quickly absorb plasma in blood. The sponge also stimulates interfacial reactions with erythrocytes and platelets. Compared to commercial gauze sponges, this hemostatic sponge demonstrated improved hemostatic efficiency and holds great potential for use in hemostatic applications, wound treatment, and targeted drug delivery in clinical settings [130].

Zheng et al. developed a novel AuNPs (gold nanoparticles) corn stalk/chitin composite sponge (CCAu). Investigations showed relevant properties, including hemostasis, antibacterial effects, and wound-healing promotion. In both in vitro and in vivo tests, the CCAu sponge demonstrated excellent hemostatic ability and biocompatibility, as well as effective antibacterial activity against *S. aureus* and *E. coli*. Additionally, the sponge was found to accelerate wound healing by promoting cell migration, angiogenesis, and collagen deposition [135].

## 4. Conclusions and Future Perspectives

Uncontrolled bleeding is a significant factor that can impact a patient’s recovery and quality of life after surgery. Managing blood loss during surgery can significantly reduce the risk of major perioperative complications. In cases of traumatic injuries, prompt and effective hemostasis is critical to prevent excessive blood loss, which can lead to shock and potentially fatal outcomes. Effective blood management strategies, including the use of appropriate hemostatic agents, can significantly reduce morbidity and mortality associated with traumatic injuries.

Chitosan’s exceptional biochemical properties make it an important polymeric biomaterial for use in biomedical applications that require hemostatic properties. It can be processed into various products, such as scaffolds and nanoparticles, which are increasingly being used in the rapidly growing field of nanomedicine. Chitosan-based composite materials can be optimized in various forms to achieve fast hemostasis. In order to obtain these composite materials, chitosan can be blended with other functional components, such as pain relievers, anti-inflammatory agents, and wound-healing materials, to obtain multi-functional CS-based composite hemostatic materials. 

Currently, there is no hemostatic product on the market that can be considered an ideal hemostatic product, but this ideal could be achieved using composite materials obtained by combining different hemostatic materials with different mechanisms of hemostasis that can lead to synergistic effects and, thus, faster hemostasis.

It is anticipated that the field of nanotechnology will continue to improve hemostatic materials by improving key properties and incorporating new functionalities. The ultimate goal is to design a hemostatic material with optimal properties that can induce rapid and effective clotting under a variety of bleeding conditions, such as patients with blood deficiencies or injuries of different sizes and shapes. To achieve this, it is important to continue developing a deeper understanding of the interactions between different nanomaterials and blood.

As medical services continue to advance, the demand for high-performance hemostatic materials is increasing. The fabrication of novel hemostatic materials that are efficient, safe, and easy to transport has become a critical research area. Despite advances in surgical techniques and procedures, uncontrolled bleeding remains an important complication that contributes to poor clinical outcomes, remaining one of the leading causes of morbidity and mortality in trauma, childbirth, and complex surgeries.

In order to develop an optimal hemostatic dressing, significant interdisciplinary research endeavors are essential. These efforts involve conducting comprehensive studies that encompass various disciplines. Key areas of focus include establishing and refining animal models of trauma, devising efficient hemostatic devices, and performing clinical trials within multidisciplinary settings. These combined research efforts are crucial for advancing the field and achieving the fabrication of an ideal hemostatic dressing.

## Figures and Tables

**Table 1:** Characteristics and hemostatic mechanism of different hemostatic materials and commercially available topical hemostatic agents.

Materials and Trademarks	Hemostatic Mechanism	Characteristics	Ref.
**Chitosan-based materials** ChitoFlex, Chitoseal, Celox, TraumaStat, HemCon.	Positive surface charge enables it to bind with negatively charged blood components, promoting platelet activation and the agglutination of blood proteins to facilitate fibrin clot formation, while also forming a strong physical barrier that adheres to wet tissues and seals wounds.	Biocompatible, biodegradable, antibacterial ability, stimulatory effect on tissue regeneration, hemostatic effect, cost-effective, easy to store, and long shelf-life; suitable for patients with coagulopathy, although it may not be entirely effective for extensively bleeding wounds.	[26,27,28,29,30]
**Cellulose-based materials** BloodSTOP, WoundClot, Surgicel, Suntouch, ActCel.	Absorbs fluids, forms a physical barrier to prevent blood loss, exhibits anti-microbial activity, is bioabsorbable, and aids clotting by binding to calcium ions, initiating the clotting cascade through contact activation and decreasing pH at the wound site, leading to platelet activation and aggregation.	Appropriate for achieving hemostasis in cases of capillary, arteriolar, venous, and bone bleeding, and is also biocompatible, non-immunogenic, and bactericidal, conforming well to the wound site. However, it may not be effective in managing severe bleeding.	[2,31,32,33,34]
**Starch-based materials** PerClot, EndoClot	Absorbs water from the blood, leading to the formation of a gel-like matrix that can adhere to tissue and promote the aggregation of platelets and the activation of clotting factors, ultimately resulting in the formation of a stable fibrin clot that can help to stop bleeding.	Reduces bleeding and the need for transfusions, minimizes the risk of blood infections, and has no known immune or allergic reactions or toxic side effects. Should not be used in blood vessels to avoid the risk of embolism and is suitable for minor injuries. It is easy to use, lightweight, has a long shelf life, and is inexpensive.	[35,36,37,38,39,40]
**Collagen-based materials** Avitene, Helitene, Hemopad, Helistat, Collastat, Instat, CoStasis, D-stat.	Forms a physical matrix, triggers the process of the coagulation cascade, and induces the activation of platelets, leading to the release of clotting factors such as thrombin and fibrinogen.	Promotes tissue regeneration and repair and possesses characteristics such as biocompatibility, cell adhesion, biodegradability, non-toxicity, and low antigenicity. Effective in heparinized patients, not suitable for use in patients with thrombocytopenia.	[2,12,26,41,42]
**Gelatin-based materials** Gelfoam, Surgifoam, Gelfilm, Gelita-spon.	Triggers the activation and aggregation of platelets, expedites the formation of clots, and provides structural support to the clot formed by enhancing thrombin generation and subsequently propagating the coagulation cascade.	Applicable for treating various types of wounds and injuries, but caution must be taken when used in restricted areas or near nerve structures due to the potential risk of compressive complications. It is a cost-effective solution that remains stable at room temperature, non-toxic, and non-antigenic. Furthermore, it has strong adsorption capability, can stick to the wound surface, and can increase its volume up to twice its original size by absorbing fluids.	[14,43,44,45,46]
**Fibrin-based materials** Artiss, Tissel, Evicel, Tissucol, TachoSil.	Major protein component of blood clots, is formed as the final step in the coagulation cascade, serving as a scaffold for tissue repair and providing cues for cell behavior during injury healing.	Exhibits excellent hemostatic and adhesive properties and biocompatibility and can be used for severe bleeding and patients with coagulation disorders, but is not recommended for application on blood vessels. It also aids in tissue regeneration following injury due to its fast polymerization dynamics and ease of tunability.	[47,48,49,50]
**Polycyanoacrylates-based materials** Dermabond, Omnex, Glubran, Histoacryl, GLUture.	Rapidly polymerizes upon contact with fluids to create a mechanical barrier or plug that occludes the bleeding vessel or tissue, resulting in hemostasis.	Presents bactericidal and bacteriostatic effects, non-toxic, non-carcinogenic, and good histocompatibility, with considerable hemostatic ability and can be applied in anastomosis, wound hemostasis, wound adhesion, and tendon repair; however, it may result in vascular embolization and the release of toxic substances.	[45,51,52,53]
**PEG-based materials** Coseal	Upon contact with tissue fluids, forms a gel-like matrix, which adheres to the tissue and provides a mechanical barrier to prevent bleeding.	Favorable biocompatibility, minimal cytotoxicity, and excellent hemostatic properties, commonly utilized in surgical settings to reduce bleeding and facilitate wound healing with a low occurrence of unfavorable consequences.	[41,54,55,56,57]
**Polyurethane-based materials** Bioclusive, Opsite Flexigrid, Tegaderm, Allevyn, Tegaderm (3M Science), TissuGlu (Cohera Medical, Inc.), ResQFoam (Arsenal Medical, Inc.), Nanosan-Sorb (SNS Nano Fiber Technology)	Triggers the activation and aggregation of thrombocytes, initiating the coagulation cascade.	Polyurethane dressings can maintain a moist wound environment by allowing the transmission of moisture, oxygen, and air while blocking fluids and bacteria and providing thermal insulation, promoting autolytic debridement; their high absorbency is due to a hydrophilic contact surface, microporous foam, and hydrophobic backing. Maintains its shape and firmness when exposed to blood,	[44,58,59,60]
**Zeolite and kaolin powder-based materials** Quikclot, Woundstat Combat Gauze	Has a hygroscopic action, which allows it to quickly absorb water from blood to concentrate coagulation factors; it can also release Ca^++^ in blood and activate FXII to trigger the intrinsic coagulation pathway and potentially induce the contact activation of platelets.	Ease of use, stability, no biological toxicity or disease transmission, provide deep tissue access, are inert, and do not elicit an immune response. Not bioabsorbable and are less effective for arterial bleeding or coagulopathic patients. Their success is dependent on the patient’s blood-clotting activity. May cause thrombotic complications if particles enter the bloodstream.	[19,41,61,62,63]

PEG—polyethylene glycol; PU—polyurethane; FXII—coagulation factor XII.

**Table 2:** Examples of commercial brands of hemostatic materials containing chitosan and their mechanism of action.

Trademark	Mechanism of Action and Characteristics
ChitoFlex	Wound dressing specifically engineered to minimize substantial bleeding by securely attaching to tissue surfaces, creating a flexible barrier that effectively seals and stabilizes the wound area. It possesses antibacterial properties and is biocompatible, ensuring compatibility with the body’s natural processes [64,65].
Chitoseal	Enhanced with a cellulose coating, this wound dressing aids in the management of hemorrhage wounds by reducing the time required for compression. It effectively stops bleeding at sites of vascular access, as well as around percutaneous catheters or tubes [64,65].
Celox	Specifically designed to address life-threatening bleeding, demonstrating remarkable effectiveness within just 30 s. Notably, it achieves hemostasis without generating heat and forms a strong plug when it comes into contact with red blood cells. Celox proves to be effective even for individuals taking antiplatelet or anticoagulant medications, as well as those experiencing hypothermia. It is available in both granular and bandage forms, with the powdered version having some limitations in certain clinical conditions [66,67].
TraumaStat	Product composed of freeze-dried chitosan that incorporates highly porous silica (acts as a robust activator of the intrinsic clotting cascade) and polyethylene. This unique composition is specifically designed for external temporary use, aiming to effectively control moderate to severe bleeding [65,67].
HemCon	A type of freeze-dried chitosan acetate salt that is primarily employed in emergency situations to halt blood loss. Its mechanism of action involves the interaction between the protonated amine groups present in chitosan molecules and the negatively charged residues on the membranes of red blood cells. This bandage has been developed in collaboration with the U.S. Army and is primarily utilized on battlefields to effectively control bleeding [65,66].

**Table 3:** Different forms of chitosan-based materials for hemostatic applications and their characteristics findings.

Forms	Composition	Characteristics	Ref.
Dressing	CS, aluminum chloride	The microporous structure of the dressing is irregular, which allows it to absorb the maximum amount of blood and promote clot formation.	[28]
	Carrageenan, CS	The composite dressing’s greater swelling, larger surface area, and mesoporous structure result in superior hemostatic activity by promoting the increased adhesion of blood cells and platelets.	[109]
	CS, calcium alginate	Biocompatibility, antibacterial, moisture retention, healing promotion, and noncytotoxicity characteristics make chitosan–calcium alginate dressing a superior option for wound care.	[110]
Hydrogel	CS, PEG	The combination of biodegradability, self-adhesiveness, self-healing ability, stretchability, antibacterial properties, and biocompatibility makes it a promising material for emergency hemostasis, particularly for joint and limb injuries. The hydrogel showed strong adhesion to various substrates (PTFE, pigskin, and glass tubes) and provided long-term stability when applied to bleeding wounds in both static and dynamic humid environments.	[111]
	Hydroxybutyl-functionalized CS	The material possesses thermosensitive characteristics, strong adhesion ability, effective hemostasis, appropriate mechanical properties, self-healing capability, easy removal as needed, antioxidant properties, as well as photothermal and intrinsic antibacterial activity.	[112]
	FCMCS, PDA, PAM	The hydrogel exhibited a variety of functions including tissue adhesion, biocompatibility, self-healing, and antibacterial properties. It also maintained its mechanical characteristics while offering broad-spectrum antibacterial activity.	[113]
	CMCS, OHA	The material exhibits favorable biodegradability and biosafety profiles, and possesses strong hemostatic and sealing capabilities, making it a promising candidate for clinical hemostatic sealant applications.	[114]
Sponge	Cs, AgNPs	The chitosan/Ag nanocomposite sponges demonstrated outstanding antibacterial activity against *Staphylococcus aureus* and *E. coli* in the antibacterial test. They also displayed good mechanical properties and noncytotoxicity, with cell viability values exceeding 90%.	[115]
	CS, cellulose	The sponge demonstrates favorable biocompatibility and hemostatic capability, making it a promising option for prompt hemostasis in cases of severe bleeding.	[116]
	CS/PVA-PD-FeO NPs	The sponge demonstrated high porosity and water absorption properties, as well as significant antibacterial activity. It facilitated gaseous exchange, absorbed wound exudate, and inhibited microbial growth in diabetic wounds. Therefore, it can be inferred that the chitosan composite sponge’s antioxidant, antidiabetic, and antibacterial properties can contribute to the healing of diabetic wounds.	[117]
	CS/AgNPs/alginate	The material demonstrated notable absorbency and a significant antimicrobial impact, particularly in assays involving *Bacillus cereus* and *Staphylococcus aureus*.	[118]
	CS, SIP	The material exhibits a strong ability to absorb fluids, as well as significant procoagulant effects, making it effective in promoting wound healing.	[119]

CS—chitosan; PEG—polyethylene glycol; CMCS—carboxymethyl chitosan; PDA—polydopamine; PAM—polyacrylamide; FCMCS—fungal mushroom-derived carboxymethyl chitosan; OHA—oxidized hyaluronic acid; AgNPs—silver nanoparticles; PD—aqueous leaf extract of Pinus densiflora; FeO—iron oxide; PVA—polyvinyl alcohol; SIP—squid ink polysaccharide.

## Data Availability

Data reported in this manuscript are available upon official request to the corresponding authors.
